# Association between length of residence and prevalence of MRSA colonization among residents in geriatric long-term care facilities

**DOI:** 10.1186/s12877-020-01885-1

**Published:** 2020-11-18

**Authors:** Teppei Sasahara, Ryusuke Ae, Akio Yoshimura, Koki Kosami, Kazumasa Sasaki, Yumiko Kimura, Dai Akine, Masanori Ogawa, Kenji Hamabata, Shuji Hatakeyama, Longzhu Cui

**Affiliations:** 1 Division of Clinical Infectious Diseases, School of Medicine, Jichi Medical University, Yakushiji 3311-1, Shimotsuke, Tochigi 329-0498 Japan; 2 Division of Public Health, Center for Community Medicine, Jichi Medical University, Yakushiji 3311-1, Shimotsuke, Tochigi 329-0498 Japan; 3 Division of Bacteriology, School of Medicine, Jichi Medical University, Yakushiji 3311-1, Shimotsuke, Tochigi 329-0498 Japan; 4 Medical corporation Sanikukai Nissin Hospital, Kiryu, Gunma 376-0001 Japan; 5 Clinical Microbiology Laboratory, Jichi Medical University Hospital, Yakushiji 3311-1, Shimotsuke, Tochigi 329-0498 Japan; 6 Health Service Center, Jichi Medical University, Yakushiji 3311-1, Shimotsuke, Tochigi 329-0498 Japan; 7 Gerontological Nursing, School of Nursing, Jichi Medical University, Yakushiji 3311-1, Shimotsuke, Tochigi 329-0498 Japan; 8 Division of Infectious Diseases, Jichi Medical University Hospital, Yakushiji 3311-1, Shimotsuke, Tochigi 329-0498 Japan

**Keywords:** Methicillin-resistant *Staphylococcus aureus* (MRSA), Older adults, Geriatric long-term care facility, Infection control and prevention

## Abstract

**Background:**

A high prevalence of methicillin-resistant *Staphylococcus aureus* (MRSA) colonization has been reported among residents in geriatric long-term care facilities (LTCFs). Some studies indicate that MRSA might be imported from hospitals into LTCFs via resident transfer; however, other studies report that high MRSA prevalence might be caused by cross-transmission inside LTCFs. We aimed to assess which factors have a large impact on the high MRSA prevalence among residents of geriatric LTCFs.

**Methods:**

We conducted a cohort study among 260 residents of four geriatric LTCFs in Japan. Dividing participants into two cohorts, we separately analyzed (1) the association between prevalence of MRSA carriage and length of LTCF residence (Cohort 1: *n* = 204), and (2) proportion of residents identified as MRSA negative who were initially tested at admission but subsequently identified as positive in secondary testing performed at ≥2 months after their initial test (Cohort 2: *n* = 79).

**Results:**

Among 204 residents in Cohort 1, 20 (9.8%) were identified as positive for MRSA. Compared with residents identified as MRSA negative, a larger proportion of MRSA-positive residents had shorter periods of residence from the initial admission (median length of residence: 5.5 vs. 2.8 months), although this difference was not statistically significant (*p* = 0.084). Among 79 residents in Cohort 2, 60 (75.9%) were identified as MRSA negative at the initial testing. Of these 60 residents, only one (1.7%) had subsequent positive conversion in secondary MRSA testing. In contrast, among 19 residents identified as MRSA positive in the initial testing, 10 (52.6%) were negative in secondary testing.

**Conclusions:**

The prevalence of MRSA was lower among residents with longer periods of LTCF residence than among those with shorter periods. Furthermore, few residents were found to become MRSA carrier after their initial admission. These findings highlight that MRSA in LTCFs might be associated with resident transfer rather than spread via cross-transmission inside LTCFs.

## Background

Admission to geriatric long-term care facilities (LTCFs) is increasingly required for frail older adults in countries with large aging populations. It is challenging to enhance the quality of daily care in these facilities. Particularly, infection prevention and control remain a considerable challenge because frail residents are vulnerable to specific infectious diseases that can cause outbreaks [1–8]. Methicillin-resistant *Staphylococcus aureus* (MRSA) is one of the most common multidrug-resistant organisms affecting mortality among residents of LTCFs [9–14]. Studies from around the world have reported a wide-ranging prevalence of MRSA colonization among LTCF residents of 4–65% [14–24]. This prevalence is much higher than that of the general population, as well as that in hospital settings [25–29], leading to the conclusion that geriatric LTCFs are potential reservoirs for MRSA [24, 29–35].

Several studies using genotyping methods have previously indicated that MRSA is most likely imported from hospitals into LTCFs via the transfer of patients [35–38], which might be associated with the high prevalence of MRSA colonization among LTCF residents. However, other studies have reported that this high prevalence is possibly caused by cross-transmission of MRSA inside LTCFs [34, 39, 40]. In the present study, we aimed to assess which factors have a large impact on the high prevalence of MRSA colonization among residents of LTCFs. We hypothesized that if MRSA spreads from resident to resident inside LTCFs, individuals with longer periods of LTCF residence would have a higher prevalence of MRSA colonization; furthermore, residents in whom MRSA is not detected at the initial LTCF admission would acquire MRSA colonization after admission to an LTCF. To test these hypotheses, we investigated the association between MRSA carriage and length of residence among LTCF residents.

## Methods

### Design, settings, and participants

We conducted a cohort study among residents receiving long-term care in four geriatric LTCFs of Japan. These facilities were selected as they are connected with specific back-up hospitals from which residents are transferred according to their medical needs. The facilities were anonymized owing to the ethics protocol followed; however, the brief profiles of the included LTCFs are listed in Table 1.

**Table 1:** Characteristics of facilities

	Geriatric long-term care facilities (anonymized)			
Characteristics	A	B	C	D
Facility type	HSF	HSF	SNH	HSF
Resident capacity	100	50	60	150
Male: Female residents	47: 53	11: 32	19: 50	33: 67
Age of residents, median (range), year	84 (59–106)	91 (77–105)	87 (70–106)	85 (53–105)
Population density of municipality where facility is located (persons/km^2^)^a^	389	54	389	13,370
Region of Japan	Eastern	Eastern	Eastern	Western
Number of beds in the back-up hospital	90	100	90	327

*Abbreviations*: *HSF* health services facility, *SNH* special nursing home

^a^ Calculated using population of the municipalities in 2019 or 2020

In Japan, geriatric LTCFs are classified according to two main types: (1) geriatric health services facilities; and (2) geriatric special nursing homes. The former are intermediate facilities between hospitals and nursing homes, with a primary focus on rehabilitation. These facilities typically have a goal of returning patients to home-based care, although some residents may require long-term care for years. Geriatric special nursing homes provide daily life support, including end-of-life care. We included three geriatric health services facilities and one special nursing home in the study.

**Fig. 1:**
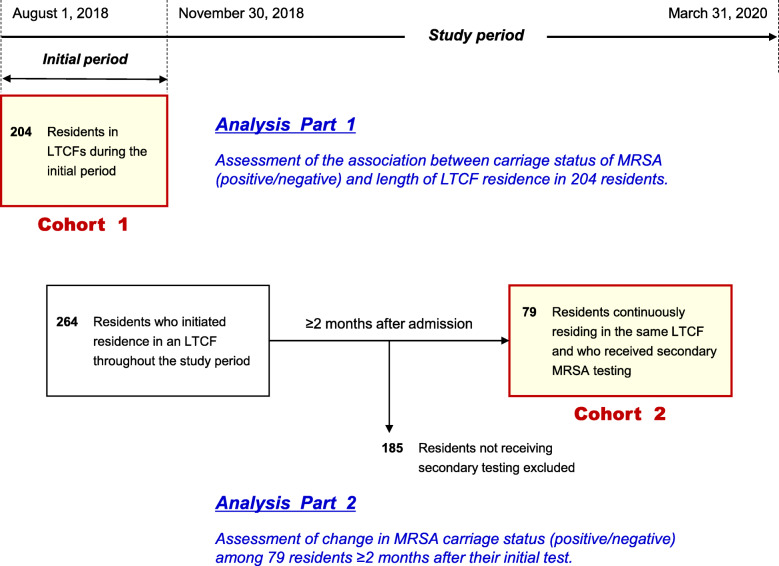
Outline of the study. Abbreviation: LTCF, long-term care facility. MRSA, methicillin-resistant *Staphylococcus aureus.* Some residents could be included in both cohorts

Among residents living in these four facilities, study participants were those underwent testing for MRSA carriage during the study period, from August 2018 through March 2020. We divided participants into two cohorts: (1) those residing in an LTCF during the initial 4-month study period (from August 2018 to November 2018), and (2) those who initiated residence in an LTCF throughout the study period (August 2018 to March 2020) (Fig. 1). Residents in Cohort 1 included those who were already residing in an LTCF before the initial study period as well as those who were initially admitted to an LTCF during the period. Therefore, some residents could be included in both cohorts.

### Microbiology and MRSA isolates

Testing for MRSA carriage was performed among residents admitted to an LTCF during the study period. Both nasal cavities of each resident were tested. Each nasal vestibule was swabbed five times, rotating the swab while exerting gentle pressure [41]. Seed-Swab MRSA™ kits (Eiken Chemical Co., Ltd., Tokyo, Japan.) were used for swabbing and transporting samples [42]. The swabs were moistened with saline solution before each specimen was taken.

Test swabs were streaked onto selective agar plates for MRSA (MDRS-K™; Kyokuto Pharmaceutical Industrial Co., Ltd., Tokyo, Japan.) and incubated for 24–48 h at 35 °C [43]. Isolates presumptively identified as MRSA based on colony morphology (mannitol positive, lecithinase-positive yellow colonies) on MDRS-K plates were confirmed as MRSA using a VITEK 2 automated system (bioMerieux, Durham, NC, USA) and oxacillin resistance was tested using the disk diffusion method.

### Statistical analyses

In the first analysis, which was focused on Cohort 1, we assessed the association between carriage status of MRSA (positive/negative) and length of LTCF residence among 204 residents. (Fig. 1, Analysis Part 1). We described the distribution of length of LTCF residence from the initial admission, according to MRSA status. Additionally, we compared the median length of residence from the initial admission, according to MRSA status, using the Mann–Whitney U test.

In the second analysis (Cohort 2), we assessed change in MRSA carriage status among residents ≥2 months after their initial MRSA test (performed ≤1 month after initial admission). For this analysis, we excluded residents who did not undergo a second MRSA test. Among residents who were identified as MRSA negative at their initial admission, we examined the proportion of residents who had converted to positive status at the secondary testing.

All analyses were performed using IBM SPSS Statistics for Windows, Version 25 (IBM Corp., Armonk, NY, USA). Categorical variables are presented as number and percentage whereas numerical variables are presented using median and interquartile range (IQR) unless otherwise indicated. The significance threshold was set at *p* < 0.05. This study was approved by the Jichi Medical University Bioethics Committee for Medical Research (Receipt ID: 20–058).

## Results

A total of 260 residents were analyzed. Cohort 1 and Cohort 2 included 204 and 79 residents in the analyses, respectively (Fig. 1). Cohort 1 comprised 93 (45.6%), 46 (22.5%), and 65 (31.9%) residents from facilities A, B, and C, respectively. No residents from facility D were included in Cohort 1 because none of these residents received MRSA testing during the initial study period. Among 204 residents in Cohort 1, 20 (9.8%) were identified as positive for MRSA (Table 2). The prevalence of MRSA carriage differed among facilities (12.9, 6.5, and 7.7% in facility A, B, and C, respectively). The median (IQR) length of residence periods was 6 (2–15) months in the Cohort 1, which included 140 (68.6%) residents with ≤1 year of residence in an LTCF.

**Table 2:** Basic characteristics of Cohort 1 (*n* = 204)

	n	(%)
MRSA test result		
Positive	20	(9.8)
Negative	184	(90.2)
Length of residence since admission, months^a^		
Minimum to maximum	1 to 114	
Median (interquartile range)	6	(2–15)
1–6	112	(54.9)
7–12	28	(13.7)
13–18	22	(10.8)
19–24	12	(5.9)
25+	30	(14.7)

*Abbreviation*: *MRSA* methicillin-resistant *Staphylococcus aureus*

^a^ Measured from admission to the time MRSA testing was performed during the initial 4-month study period (August 2018 to November 2018)

**Fig. 2:**
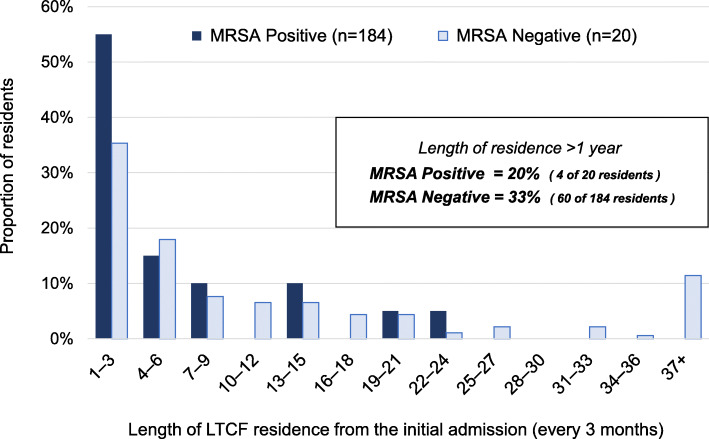
Resident distributions for length of LTCF residence from the initial admission, according to MRSA colonization (*n* = 204). Abbreviation: LTCF, long-term care facility. MRSA, methicillin-resistant *Staphylococcus aureus*

**Fig. 3:**
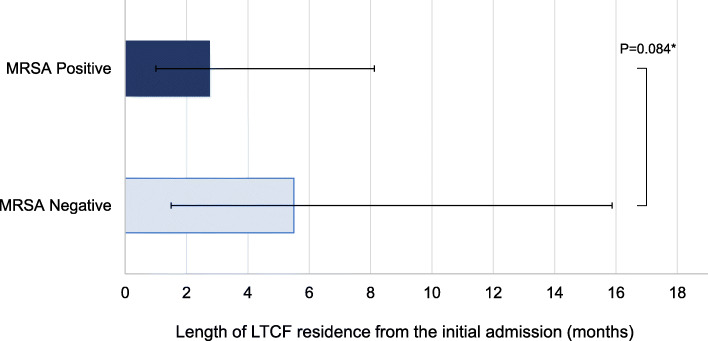
Median with interquartile range of LTCF residence length from the initial admission, according to MRSA status (*n* = 204). Abbreviation: LTCF, long-term care facility. MRSA, methicillin-resistant *Staphylococcus aureus.* * Mann-Whitney U test

Compared with residents identified as MRSA negative, there was a larger proportion of residents with 1–3 months’ LTCF residence since initial admission and MRSA-positive status (35.3% vs 55.0%) (Fig. 2). Fewer residents with > 1 year LTCF residence were MRSA positive than MRSA negative (20% vs 33%). The median length of residence was shorter among residents identified as MRSA positive than those who were MRSA negative, although a marginally significant association was found between these groups (2.8 months vs 5.5 months; *p* = 0.084) (Fig. 3). These results indicate that residents with shorter periods of LTCF residence were more likely to be MRSA carriers whereas those with longer periods of residence were less likely to have MRSA colonization.

All residents included in Cohort 2 received secondary MRSA testing ≥2 months after their initial test: the median (IQR) testing interval was 3 (2.5–5.5) months (Table 3). Cohort 2 comprised 16 (20.3%), 9 (11.4%), 15 (19.0%), and 39 (49.4%) residents from facilities A, B, C, and D, respectively. Of 79 residents, 19 (24.1%) were identified as MRSA positive in their initial test. Among 60 residents identified as MRSA negative in the initial testing, only one (1.7%) resident subsequently showed positive conversion in the second test (Table 4). In contrast, among 19 residents identified as MRSA positive in the initial testing, 9 (47.4%) remained positive in secondary testing.

**Table 3:** Basic characteristics of Cohort 2 (*n* = 79)

	n	(%)
Initial MRSA test result^a^		
Positive	19	(24.1)
Negative	60	(75.9)
Second MRSA test result^b^		
Positive	10	(12.7)
Negative	69	(87.3)
Interval from initial to secondary MRSA testing, months		
Median (interquartile range)	3	(2.5–5.5)

*Abbreviation*: *MRSA* methicillin-resistant *Staphylococcus aureus*

^a^ Performed at ≤1 month after residents’ initial admission

^b^ Performed ≥2 months after residents’ initial test

**Table 4:** Conversion of MRSA status in Cohort 2 (*n* = 79)

	Initial MRSA test result^a^			
	Positive (*n* = 19)		Negative (*n* = 60)	
	n	(%)	n	(%)
Second MRSA test result^b^				
Positive (*n* = 10)	9	(47.4)	1	(1.7)
Negative (*n* = 69)	10	(52.6)	59	(98.3)

Abbreviation: *MRSA* methicillin-resistant *Staphylococcus aureus*

^a^ Performed at ≤1 month after residents’ initial admission

^b^ Performed ≥2 months after residents’ initial test

## Discussion

Given that MRSA can frequently spread inside geriatric LTCFs via cross-transmission among residents, those with longer periods of residence are considered to have a higher risk for MRSA colonization, which would result in a higher prevalence of MRSA. However, our findings conversely indicated that the prevalence of MRSA was lower among residents with longer residence periods than those with shorter periods. Furthermore, among residents living in LTCFs ≥2 months from their initial admission, only 1 in 60 was identified as having positive conversion to MRSA colonization whereas about half had negative conversion. Findings from previous studies remain controversial as to whether the high prevalence of MRSA among LTCF residents is mainly owing to importation from outside the facility via admission of residents who are colonized with MRSA or whether the high prevalence is owing to cross-transmission inside of LTCFs [33, 34, 39, 40]. Our findings indicated that MRSA might be carried into the LTCFs via transfer of residents rather than spread via cross-transmission inside the LTCFs.

Previous studies have reported that among LTCF residents, 20–50% are potentially persistent nasal MRSA carriers, but about 50% of these residents exhibit negative conversion with time [23, 40]. Another article also indicated that MRSA within the human nasal cavity can disappear over time [22], which is consistent with our results. If residents indeed acquire MRSA via transmission from persistent carriers inside LTCFs, those with longer periods of LTCF residence would be at greater risk for MRSA acquisition, which would prove our hypothesis that LTCF residents with longer periods of residence have a higher prevalence of MRSA. Some previous studies have assumed that geriatric LTCFs are potential reservoirs for MRSA because of the high MRSA prevalence in these facilities [24, 29–35]. However, our study indicated that admission of residents with nasal MRSA colonization might be a primary contributor to the high prevalence of MRSA among LTCF residents.

There are two possible routes via which MRSA may be introduced to LTCFs from outside the facility. The main route would likely be importation from hospitals, which is largely supported by the results of previous studies [35–38]. In addition to these reports, one study previously found that LTCFs with a larger number of hospitals located nearby had a higher prevalence of MRSA than LTCFs with fewer hospitals nearby [44]. Another possible route is via the general population [45]. The prevalence of MRSA in the Japanese general population is estimated to be ≤5% [46–48].; however, this prevalence might be higher among older adults owing to frailty, in comparison with younger people [49]. Furthermore, regional MRSA epidemics of community-acquired strains have recently occurred in Japan, which might affect MRSA prevalence [45, 48, 50, 51]. A previous study found regional differences in the MRSA prevalence [44, 52]. Our results showed that the prevalence of MRSA differed among LTCFs, which might reflect differences in the MRSA prevalence among the general population where each LTCF is located.

Care providers in geriatric LTCFs should consider that frail residents who are initially admitted to the facility are most likely to have nasal MRSA colonization, especially those admitted from hospitals. Nasal application of mupirocin for residents identified as having MRSA at the initial admission might be effective for the prevention of MRSA transmission inside an LTCF; however, this should be carefully assessed owing to the high costs as well as the possible increase in MRSA with resistance to mupirocin [53]. Universal precautions against MRSA transmission should therefore be appropriately applied by all LTCF staff. Among various prevention strategies, thorough hand hygiene as well as appropriate use of gloves is recommended to maximize deficient medical resources in LTCFs and to reduce excess costs [54, 55]. Enhanced efforts to prevent the development of decubitus ulcers [56] and wearing gowns and gloves when caring for residents with indwelling devices, such as a feeding tube or urinary catheter [6], may also be effective in preventing MRSA transmission inside an LTCF. Furthermore, a patient traceability system could be establisehed, with an alarm system between specific back-up hospitals where colonization is alerted and the receiving LTCFs.

This study includes some limitations. First, we did not obtain complete information on the background of residents, such as sex, age, general condition, and medical history owing to the ethics protocol followed; the ethics review board did not grant approval to obtain this information. Among these factors, general status and medical history, including comorbidities, nutritional status, and duration of prior hospitalization, might affect the prevalence of MRSA [49]. Second, we could not obtain information on the prevalence of MRSA carriage in specific regional general populations around each LTCF, as well as among patients hospitalized in each specific back-up hospital, which might affect our results. Third, our study participants included a small number of residents from limited LTCFs. A larger number of residents from a greater number of LTCFs located in both rural and urban regions is required, to further confirm our findings. Fourth, we performed MRSA testing only twice after admission. Multiple tests with longer follow-up might be required to accurately assess the status of MRSA colonization. Fifth, we could not obtain information regarding from where residents had been transferred to the LTCFs. Sixth, not all residents initially admitted to the LTCFs were included in Cohort 2 because some did not agree to undergo secondary MRSA testing. Seventh, we only performed nasal MRSA testing. Some residents have MRSA on their skin or in wound sites, which might result in a higher prevalence. Finally, we did not exclude residents from the analysis who had previously been discharged from an LTCF but who were subsequently readmitted.

## Conclusions

The prevalence of MRSA was lower among residents with longer periods of residence in an LTCF than in those with shorter residence periods. Furthermore, few residents were identified as having positive conversion to MRSA colonization after their initial admission. These findings highlight that MRSA might be carried into LTCFs via the transfer of new residents rather than spread via cross-transmission inside LTCFs. Residents with recent admission to LTCFs might have a large impact on the high prevalence of MRSA.

## Data Availability

The datasets used and/or analyzed during the current study are available from the corresponding author on reasonable request.

## Abbreviations

MRSA: Methicillin-resistant *Staphylococcus aureus*
LTCF: Long-term care facility
IQR: Interquartile range
